# Early functional maturation of anti-Spike antibodies predicts efficient SARS-CoV-2 RNA clearance

**DOI:** 10.3389/fimmu.2026.1948045

**Published:** 2026-10-02

**Authors:** Danielle A. S. Rodrigues, Barbara Gabrielle de Araujo, Julia Pestana, Layla de Freitas, Brenda N. Ramos, Lendel C. da Costa, Matheus V. Andrade, Letícia M. Raposo, Vicente B. T. Bozza, Luciana Conde, Elena Montes-Cobos, Andreza M.D.S. Gama, Vinicius M. Vidal, Rafael M Galliez, Amilcar Tanuri, Orlando D. C. Ferreira, Marcus Vinicius de Mattos Silva, Ronaldo Mohana-Borges, Leda R. Castilho, Juliana Echevarria-Lima, Luiza M. Higa, Terezinha M. Castineiras, Marcelo T. Bozza, André M. Vale

**Affiliations:** 1 Laboratório de Biologia de Linfócitos, Instituto de Biofísica Carlos Chagas Filho, Universidade Federal do Rio de Janeiro, Rio de Janeiro, RJ, Brazil; 2 Laboratório de Virologia Molecular, Departamento de Genética, Instituto de Biologia, Universidade Federal do Rio de Janeiro, Rio de Janeiro, RJ, Brazil; 3 Centro de Ciências Exatas e Tecnologia, Universidade Federal do Estado do Rio de Janeiro, Rio de Janeiro, RJ, Brazil; 4 Instituto de Microbiologia Paulo de Góes, Universidade Federal do Rio de Janeiro, Rio de Janeiro, RJ, Brazil; 5 Núcleo de Enfrentamento e Estudos de Doenças Infecciosas Emergentes e Reemergentes, Universidade Federal do Rio de Janeiro, Rio de Janeiro, RJ, Brazil; 6 Laboratório de Biotecnologia e Bioengenharia Estrutural, Instituto de Biofísica Carlos Chagas Filho, Universidade Federal do Rio de Janeiro, Rio de Janeiro, RJ, Brazil; 7 Laboratório de Engenharia de Cultivos Celulares, Programa de Engenharia Química, COPPE, Universidade Federal do Rio de Janeiro, Rio de Janeiro, RJ, Brazil

**Keywords:** antibody kinetics, cross-reactivity, neutralizing antibodies, SARS-CoV-2, vaccination, viral persistence

## Abstract

**Introduction:**

Viral RNA clearance following mild SARS-CoV-2 infection varies considerably among individuals, but the early humoral immune features underlying this heterogeneity remain incompletely understood.

**Methods:**

We investigated whether the kinetics and functional maturation of the antibody response were associated with viral RNA clearance in a longitudinal study of 78 healthcare professionals infected with the ancestral Wuhan SARS-CoV-2 strain and followed for up to three years. Participants were stratified according to viral RNA clearance kinetics as nonpersistent (≤21 days) or persistent (>21 days). Humoral responses were longitudinally characterized by seroconversion kinetics, antibody isotypes and IgG subclasses, affinity, cross-variant recognition, and neutralizing activity.

**Results:**

Nonpersistent individuals exhibited accelerated seroconversion to Spike and receptor-binding domain antigens within the first 11 days after symptom onset, accompanied by significantly greater neutralizing activity against the ancestral strain. In contrast, persistent individuals displayed delayed seroconversion, prolonged IgM responses, and reduced coordination among IgG subclasses during acute infection, consistent with delayed early humoral maturation. Total immunoglobulin levels did not distinguish the groups, whereas cross-variant antibody recognition during acute infection remained limited and predominantly strain-focused in both groups. During convalescence, durable anti-Spike IgG responses were maintained independently of persistence status, without significant differences in cross-variant breadth. Vaccination robustly enhanced antibody titers, variant recognition, and ACE2-blocking responses in both groups.

**Discussion:**

Accelerated functional maturation of the early anti-Spike antibody response, rather than overall antibody magnitude, was associated with efficient SARS-CoV-2 RNA clearance. These findings identify early functional maturation of the humoral response as an immunological correlate of efficient viral clearance and provide mechanistic insight into the immune processes underlying heterogeneous SARS-CoV-2 RNA clearance during mild infection.

## Introduction

The coronavirus disease 2019 (COVID-19) emerged in December 2019 as a novel infectious disease caused by severe acute respiratory syndrome coronavirus 2 (SARS-CoV-2) and rapidly evolved into a global pandemic, resulting in millions of deaths worldwide (1, 2). SARS-CoV-2 infection displays marked heterogeneity in both clinical presentation and virological kinetics, ranging from asymptomatic or mild disease to severe respiratory failure (3). In most mild cases, replication-competent virus is no longer detected approximately 9 days after symptom onset; however, viral RNA may remain detectable by RT-qPCR for 14–17 days or longer (4). Prolonged RT-qPCR positivity has been reported even in non-severe infections (5, 6), raising important questions regarding the immunological mechanisms that govern viral RNA persistence and clearance in the respiratory tract.

Although prolonged viral RNA detection in mild disease is not typically associated with worse clinical outcomes, it may reflect differences in host immune dynamics. Early viral control depends on coordinated innate and adaptive immune responses, including timely activation of virus-specific T and B cell compartments (7, 8). In particular, humoral immunity plays a central role in limiting viral spread by neutralizing virions and facilitating Fc-mediated effector functions (9). Neutralizing antibodies targeting the Spike (S) glycoprotein, especially the receptor-binding domain (RBD), have been established as key correlates of protection against symptomatic infection and reinfection (10, 11). However, antibody quantity alone does not fully explain variability in infection outcomes, suggesting that qualitative features such as affinity, subclass distribution, and timing of induction may critically influence viral clearance.

In the context of acute SARS-CoV-2 infection, immunological memory in the form of circulating antibodies and memory B cells persists for at least eight months after symptom onset (12). Longitudinal studies have demonstrated ongoing antibody evolution and increasing neutralization potency months after primary infection, even in the absence of re-exposure (13, 14). These observations underscore the dynamic nature of humoral maturation following SARS-CoV-2 infection and highlight the importance of affinity maturation and clonal evolution in shaping long-term immunity.

Humoral responses to SARS-CoV-2 are predominantly mediated by immunoglobulin G (IgG), particularly the IgG1 subclass and, to a lesser extent, IgG3, with additional contributions from IgG2 and IgG4, as well as IgA and IgM isotypes (15, 16). IgA is particularly relevant to antiviral immunity at mucosal sites, where it contributes to local immune defense, whereas early IgM responses typically precede class switching. IgG1 and IgG3 generally predominate in systemic antiviral response and have been associated with Th1-biased inflammatory environments (17). IgG2 and IgG4 can also be induced following SARS-CoV-2 infection, although their early induction has been associated with less functional humoral responses in severe COVID-19 (15). Vaccination studies have shown that the initial IgG response after a single dose is largely characterized by IgG1 and IgG3, while booster immunization increases overall IgG levels and promotes subclass diversification (18, 19). Germinal center–dependent maturation processes are central to the generation of high-affinity, class-switched antibodies and durable humoral memory (20, 21), yet the kinetics of these processes during early infection, and their relationship to viral RNA clearance, remain incompletely defined.

The emergence of SARS-CoV-2 variants of concern further complicated the landscape of protective immunity. Primary infection with the ancestral Wuhan strain generally induces strain-focused neutralizing responses, and several variants demonstrate partial escape from antibodies elicited by earlier strains (22, 23). Repeated antigen exposure through infection or vaccination can enhance neutralization breadth and expand cross-reactive memory B cell clones (24). However, it remains unclear whether differences in viral persistence during primary infection influence the maturation kinetics or long-term qualitative features of the antibody response.

Despite extensive characterization of humoral responses in SARS-CoV-2 infection, few studies have directly examined how early antibody kinetics and functional quality relate to the duration of viral RNA detection in mild cases. In particular, it remains uncertain whether accelerated generation of high-affinity and neutralizing antibodies contributes to rapid viral clearance, or whether delayed humoral maturation underlies prolonged viral RNA detection.

In our previous longitudinal analysis of a cohort of healthcare professionals from the same population infected during the first epidemic wave, prolonged viral RNA detection was associated with reduced circulating plasmacytoid dendritic cells (pDCs) and natural killer (NK) cells, impaired systemic type I interferon responses, a shift from type 1 toward type 3 inflammation, and differences in anti-Spike antibody binding (25). However, the functional implications of these humoral differences and their relationship to viral clearance kinetics were not fully explored. To address these questions, we performed a comprehensive longitudinal analysis of humoral immune responses in healthcare professionals infected during the first epidemic wave with the ancestral Wuhan strain. By stratifying individuals according to viral RNA clearance kinetics, we investigated how the timing, affinity, subclass distribution, neutralization capacity, and cross-variant recognition of SARS-CoV-2–specific antibodies relate to viral persistence and subsequent immune architecture. Our findings demonstrate that accelerated functional maturation of the early anti-Spike antibody response, characterized by the generation of high-affinity, neutralizing antibodies, is strongly associated with rapid SARS-CoV-2 RNA clearance, whereas delayed humoral maturation characterizes viral RNA persistence without necessarily conferring a measurable advantage in long-term cross-variant immunity.

## Material and methods

### Study cohort

This longitudinal cohort study enrolled 78 healthcare professionals (54 women and 24 men; mean age, 42 years; IQR, 35–49.5) diagnosed with mild to moderate COVID-19. Blood plasma and nasopharyngeal swab samples were collected between March 2020 and February 2023, encompassing the Ancestral, Gamma, Delta, and Omicron BA.1 waves, as well as the vaccination rollout (started in January 2021). All participants were infected with the ancestral Wuhan strain in March 2020. Recruitment and follow-up were conducted at the COVID-19 Diagnostic Center of NEEDIER (Núcleo de Enfrentamento e Estudos de Doenças Infecciosas Emergentes e Reemergentes) of the Federal University of Rio de Janeiro. Participants underwent serial clinical and virological follow-up until SARS-CoV-2 RNA clearance. After RT-qPCR negativity, subsequent samples were collected as part of the longitudinal study follow-up. Clinical and demographic data, including symptoms at disease onset (e.g., cough, headache, adynamia, and fever) and comorbidities (e.g., hypertension, diabetes, and autoimmune diseases), were recorded at baseline. Negative controls comprised PCR-negative healthy individuals sampled in 2020 (*n* = 59) and healthy pre-pandemic donors sampled in 2018 (*n* = 94), recruited from the blood donor bank of the State Hematology Institute Hemorio, as previously described (26).

### Viral RNA detection and persistence stratification

Total viral RNA was isolated from nasopharyngeal swabs using the Maxwell 16 Viral Total Nucleic Acid Purification Kit (Promega) according to the manufacturer’s protocol. SARS-CoV-2 detection was performed using the CDC 2019-nCoV RT-qPCR assay (Integrated DNA Technologies, IA, USA), targeting the N1 and N2 regions of the nucleocapsid gene, with human RNase P serving as an internal control. Reactions were run in parallel on a 7500 Real-Time PCR System (Applied Biosystems, CA, USA). Samples were considered positive when both viral targets amplified with cycle threshold (Ct) ≤ 40 and negative when neither target amplified or amplification occurred with Ct > 40. Participants were stratified based on the duration of viral RNA detection after symptom onset (DASO) into two groups: persistent (*n* = 43; PCR-positive for > 21 days) and nonpersistent (*n* = 35; PCR-positive for ≤ 21 days). The first PCR-negative sample collected after viral clearance was defined as the peri-infection phase. The late phase was defined as the period >70 DASO, following viral clearance. This late phase was specifically selected to evaluate the establishment and durability of long-term humoral immune memory prior to the administration of any SARS-CoV-2 vaccines.

### S-UFRJ ELISA for antibodies detection

Antigen-specific antibody responses (IgM, IgA, IgG, and IgG1–4 subclasses) against SARS-CoV-2 Spike (S), Receptor-Binding Domain (RBD), and Nucleocapsid (N) proteins were measured by S-UFRJ ELISA as described previously (26). High-binding 96-well ELISA microplates (Corning) were coated with 50 µL per well of coating solution and incubated overnight at room temperature (RT). The coating solution contained SARS-CoV-2 Spike (S), Receptor-Binding Domain (RBD), or Nucleocapsid (N) proteins diluted in PBS (Phosphate-buffered saline solution, Gibco) at a final concentration of 4μg/mL. The antigens used in this study included the SARS-CoV-2 Spike protein (SLECC1105), the Wuhan RBD protein, S and RBD proteins corresponding to SARS-CoV-2 variants, and the Nucleocapsid protein (N45KDa). The Spike protein was produced as previously described (26, 27), whereas the Nucleocapsid protein was produced in-house (28). The Wuhan RBD protein was kindly provided by Dr. John Pak (Chan Zuckerberg Biohub), and RBD variant proteins were purchased from Sino Biological Inc. After coating, the wells were washed and blocked with 150 µL of PBS containing 1% BSA (Bovine serum albumin, Sigma) (blocking solution) for 1h at RT. Following blocking, 50 µL of serum or plasma samples diluted 1:40 in PBS-1% BSA were added to each well and incubated for 2 h at RT. Plates were washed five times with 150 µL of PBS. Next, 50 µL of HRP-conjugated goat anti-human secondary antibody specific for IgG (Fc) (Cat. 2081-05), IgA (Cat. 2053-05), IgM (Cat.2023-05), and IgG subclasses 1–4 (IgG1, Cat. 9054-05; IgG2, Cat.9060-05; IgG3, Cat.9210-05; IgG4, Cat. 9200-05), all from Southern Biotech and diluted 1:8.000 in PBS-1% BSA, were added and incubated for 2h at RT. Plates were again washed five times with 150 µL of PBS. The reaction was developed with 50 µL of TMB substrate (3, 3’, 5, 5’-tetramethylbenzidine; Scienco, Brazil) and stopped by adding 50 µL of 1N HCl. Optical density (O.D.) was measured at 450 nm with background correction at 655 nm using a microplate reader (Bio-Rad). Results are expressed as the ratio between the sample O.D. and the cut-off value. The cut-off (C.O.) was defined as the mean O.D. of the negative controls on the same plate plus one standard deviation, determined from negative control samples tested under identical conditions. Samples with absorbance values below the C.O. were classified as negative, while those with values above the C.O. were considered positive.

### Plaque reduction neutralization test

Plaque Reduction Neutralization Test (PRNT) was performed for the evaluation of neutralizing antibodies against SARS-CoV-2. Serum samples were heat-inactivated at 56 °C for 30 min, followed by two-fold serial dilutions. The diluted sera were incubated with 100 plaque-forming units (PFU) of SARS-CoV-2 lineage B isolate (SP1, GenBank accession number MT126808.1) or SARS-CoV-2 Omicron lineage BA.1 isolate (EPI_ISL_7699344), for 1 h at 37 °C. In addition, virus-only and cell-only controls were included to determine virus infectivity and to monitor cell monolayer integrity, respectively. The virus-serum mixtures were inoculated onto confluent VERO-hACE2 cells seeded in 24-well plates and allowed to adsorb for 1 h at 37 °C. After adsorption, the inoculum was removed and replaced with an overlay medium (1.25% carboxymethylcellulose in alpha-MEM supplemented with 1% fetal bovine serum). Cells were further incubated for 3 days, after which the cells were fixed with 4% formaldehyde and stained with 0.25% crystal violet in 20% ethanol for plaque visualization. The plaque reduction neutralization titer was defined as the highest serum dilution resulting in a 50% reduction in plaque counts (PRNT_50_) when compared to the mean plaque counts of virus-only controls. A PRNT_50_ titer of ≥ 20 was considered positive for the presence of neutralizing antibodies (29, 30). All experiments involving replication-competent SARS-CoV-2 were conducted in a Biosafety Level 3 (BSL-3) laboratory located in the Laboratório de Virologia Molecular, Universidade Federal do Rio de Janeiro (UFRJ, Brazil).

### Multiplex surrogate neutralization assay (ACE2-blocking)

To evaluate the functional capacity of antibodies to inhibit the Spike-ACE2 interaction, sera were analyzed using the Bio-Plex Pro Human SARS-CoV-2 Neutralization Antibody Assay (Bio-Rad, Cat. 12016897), according to the manufacturer’s instructions. This competitive multiplex assay measures neutralizing antibodies that block the binding of the viral antigens to the human ACE2 receptor. Only ACE2-blocking activity against the Spike 1 (S) protein of the ancestral Wuhan strain was considered in the present analysis. Samples included in this analysis were selected based on sample availability and sufficient serum volume, without additional clinical or immunological selection criteria. In this study, ACE2-blocking activity was used as an indirect estimate of antibody affinity, with greater competitive blocking interpreted as higher inferred affinity.

### Statistical analysis

Statistical analyses were performed using GraphPad Prism version 8.0 (GraphPad Software, San Diego, CA, USA) and RStudio software. Continuous variables, including relative antibody levels, immunoglobulin concentrations, PRNT_50_ titers, and Ct values, were compared between two independent groups using the Mann–Whitney U test. Repeated-measures and longitudinal data were analyzed using a mixed-effects model (REML) followed by Tukey’s multiple comparisons test. When appropriate, comparisons among multiple independent groups were performed using the Kruskal–Wallis test followed by Dunn’s multiple comparisons post-test. Differences in categorical variables, including seropositivity rates, were assessed using Fisher’s exact test. Correlations between antigen-specific antibody responses and IgG subclasses were evaluated using Pearson’s correlation coefficient. Longitudinal antibody kinetics were modeled using locally weighted scatterplot smoothing (LOWESS) regression. The number of subjects analyzed in each group (*n*), the statistical tests applied, and the corresponding summary statistics are provided in the relevant figure legends.

## Results

### Cohort characterization and definition of viral persistence

The study cohort comprised 78 healthcare professionals (54 women and 24 men) diagnosed with mild to moderate COVID-19, all infected with the ancestral Wuhan strain in March 2020, and followed longitudinally for three years. Participants were recruited at the COVID-19 Diagnostic Center of NEEDIER (Núcleo de Enfrentamento e Estudos de Doenças Infecciosas Emergentes e Reemergentes), Federal University of Rio de Janeiro, where nasopharyngeal swabs and plasma samples were collected for SARS-CoV-2 RNA detection and antibody response measurements, respectively. A total of 369 samples were collected, representing 2–9 time points per participant, spanning from 1 to 1100 days after symptom onset (DASO). Sampling covered phases of active infection (PCR+), convalescence (PCR–), and vaccination (**Figures 1A, B**). Two control groups were included: a pre-pandemic control group (n = 94; samples collected in 2018) and a pandemic negative control group (n = 59; RT-qPCR negative and seronegative for Spike- and RBD-specific IgM, IgA, and IgG) (**Figure 1C**).

**Figure 1:**
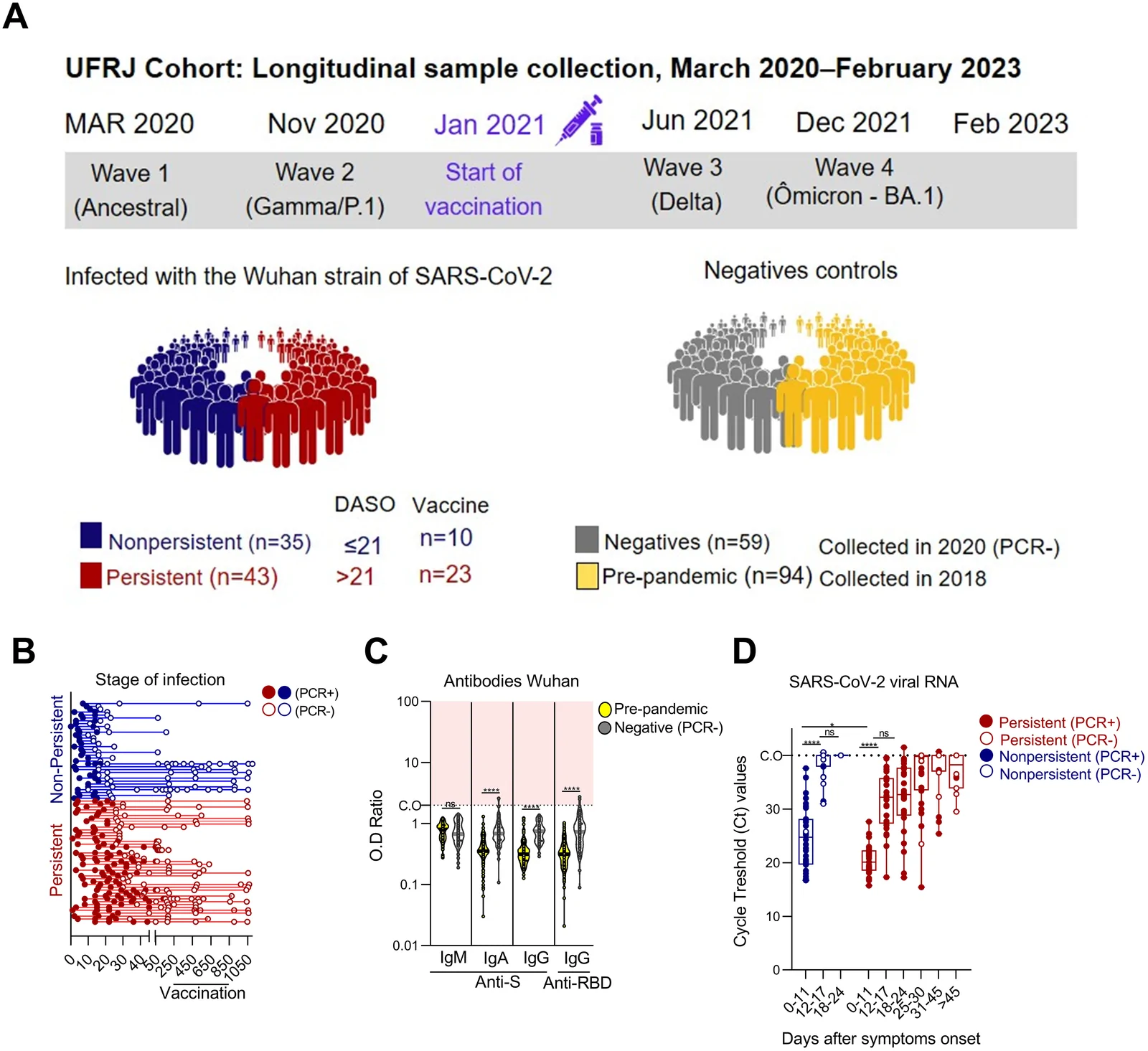
Study design and classification of viral persistence in the UFRJ SARS-CoV-2 cohort. **(A, B)** Timeline and study design of the UFRJ cohort, illustrating the longitudinal collection of blood plasma and nasopharyngeal swab samples from March 2020 to February 2023. The timeline encompasses the predominant SARS-CoV-2 variant waves (Ancestral, Gamma/P.1, Delta, and Omicron BA.1) and the onset of the vaccination campaign in January 2021. Individuals previously infected with the ancestral Wuhan strain were stratified into persistent (n = 43, red) and nonpersistent (n = 35, blue) groups based on viral RNA persistence, as defined by DASO, with cutoffs of >21 days and ≤21 days, respectively. Samples were further categorized according to longitudinal PCR status (filled circles=positive; open circles=negative). **(C)** Negative controls comprised PCR-negative individuals sampled in 2020 (n = 59, gray) and pre-pandemic donors sampled in 2018 (n = 94, yellow), all lacking SARS-CoV-2-specific immunoglobulins. **(D)** Cycle threshold (Ct) values for SARS-CoV-2 target genes. Samples with Ct ≤ 40 were considered positive, whereas those with Ct > 40 were considered negative). Statistical comparisons by Mixed-effects model (REML) test followed by Tukey post-test, Mann-Whitney U test. Bars indicate the statistical significance: ns (not significant); *(p < 0.05); ****(p < 0.0001).

SARS-CoV-2 infection was monitored through serial nasopharyngeal sampling until viral RNA clearance. Participants were monitored weekly until they achieved negative RT-qPCR results (Cycle Threshold, Ct > 40, indicating no detectable viral RNA). Based on previous NEEDIER-UFRJ data reporting a median viral positivity of 21 DASO (6), this threshold was used to define viral clearance. Individuals were thus classified as *nonpersistent* (n = 35; viral clearance ≤21 DASO) or *persistent* (n = 43; remained positive >21 DASO) (**Figure 1A**; **Table 1**). During the early phase of infection (0–11 DASO), both groups exhibited low Ct values (~20–30), indicating substantial viral RNA detection. However, Ct values were significantly lower in persistent than in nonpersistent individuals, consistent with a greater early viral RNA burden in the persistent group (**Figure 1D**). By 21 DASO, all nonpersistent participants had cleared the virus (Ct > 40), whereas some persistent individuals remained PCR positive, with Ct values ranging from 25–35 for several weeks and, in some cases, detectable RNA fragments and/or viral particles up to ~120 DASO (**Figure 1D**) (6).

**Table 1:** Baseline characteristics of study participants enrolled during the first epidemic wave (March 2020), stratified by SARS-CoV-2 RNA persistence status.

Characteristic	Overall	Nonpersistent	Persistent	*p*
	n = 78	n = 35	n = 43	
Age (years), mean ± SD	42.63 ± 9.86	43 ± 10	43 ± 10	0.931
Median (IQR)	42.5 (35–49.5)	43 (35–50.5)	41 (36–48)	
Female (%)	54 (69.23)	25 (71.43)	29 (67.44)	0.583
Male (%)	24 (30.76)	10 (28.57)	14 (32.56)	0.650
Symptoms*^†^*				
Fever (%)	46 (59.74)	20 (58.82)	26 (60.47)	>0.999
Cough (%)	59 (76.62)	25 (73.53)	34 (79.07)	0.597
Sore throat (%)	39 (50.65)	14 (41.18)	25 (58.14)	0.171
Dyspnea (%)	19 (24.68)	6 (17.65)	13 (30.23)	0.288
Headache (%)	55 (71.43)	22 (64.71)	33 (76.74)	0.312
Runny nose (%)	36 (46.75)	14 (41.18)	22 (51.16)	0.491
Nasal congestion (%)	38 (49.35)	13 (38.24)	25 (58.14)	0.109
Sneeze (%)	33 (42.86)	11 (32.35)	22 (51.16)	0.110
Ageusia (%)	39 (50.65)	20 (58.52)	19 (44.19)	0.253
Anosmia (%)	32 (41.56)	15 (44.12)	17 (39.53)	0.817
Vomiting (%)	5 (6.49)	2 (5.88)	3 (6.98)	>0.999
Nausea (%)	13 (16.88)	7 (20.59)	6 (13.95)	0.547
Diarrhea (%)	22 (28.57)	8 (23.53)	14 (32.56)	0.452
Myalgia (%)	43 (55.84)	19 (55.88)	24 (55.81)	>0.999
Adynamia (%)	49 (63.64)	23 (67.65)	26 (60.47)	0.634
Symptom duration (days) *^‡^*				
Median (IQR)	14 (6.5-20.5)	7 (4-13.75)	20 (15-30)	<0.0001
Medical history				
Hypertension (%)	12 (15.58)	3 (8.82)	9 (20.93)	0.209
Diabetes (%)	11 (14.29)	6 (17.65)	5 (11.63)	0.522
Inflammatory/autoimmune diseases (%)	14 (18.18)	6 (17.65)	8 (18.60)	>0.999
None (%)	34 (44.16)	16 (47.06)	18 (41.86)	0.817
Others (%)	27 (35.06)	10 (29.41)	17 (39.53)	0.471

*Pre-pandemic (n = 94) and PCR-negative (n = 59) controls included healthy adults.

^†^ Symptom data were available for 77 participants (34 nonpersistent and 43 persistent).

^‡^ Symptom duration data were available for 75 participants (34 nonpersistent and 41 persistent).

At disease onset, the most frequent signs and symptoms were cough (76.6%), headache (71.4%), adynamia (63.6%), and fever (59.7%). Sex, age, and comorbidity distributions including hypertension, diabetes, and autoimmune disease were comparable between groups (p > 0.05) (**Table 1**), indicating that prolonged viral RNA detection was not associated with baseline clinical characteristics in this cohort. Clinical information, including the presence and duration of symptoms, was collected during follow-up visits. Symptom duration was significantly longer in persistent than in nonpersistent individuals (median [IQR], 20 [15–30] vs. 7 [4–13.75] days, respectively; p < 0.0001) (**Table 1**). However, long-term symptoms were not systematically assessed after this initial period.

### Nonpersistent infection is associated with accelerated early seroconversion

To investigate why some individuals rapidly cleared SARS-CoV-2 RNA while others exhibited prolonged RT-qPCR positivity, we analyzed the kinetics of antigen-specific antibody responses during the early phase of infection. We focused particularly on the 0–11 DASO, a critical window for determining subsequent viral clearance dynamics. Although total IgM and IgG levels were comparable between groups (**Supplementary Figures 1A, B**), marked differences emerged in the timing and magnitude of antigen-specific responses. Most nonpersistent individuals mounted detectable and earlier IgM, IgA, and IgG responses against Spike (S), receptor-binding domain (RBD), and nucleocapsid (N) within the first 11 DASO (**Figures 2A–D**; **Supplementary Figure 1C**). In contrast, persistent individuals exhibited markedly delayed seroconversion rates, with anti-S and anti-RBD antibodies becoming detectable predominantly between 12–17 DASO, and anti-N responses emerging even later, around 18–24 DASO (**Figures 2E–H**; **Supplementary Figure 1D**). Nonpersistent individuals who developed anti-S IgM responses during the early window displayed significantly lower viral loads (i.e., higher Ct values) than persistent counterparts (**Figure 2I**). A similar pattern was observed for anti-S, anti-RBD, or anti-N IgG responses, with early IgG positivity in nonpersistent individuals being associated with higher Ct values than in persistent individuals (**Figures 2J–L**; **Supplementary Figure 1E**). Together, these results suggest that early seroconversion against key SARS-CoV-2 antigens in nonpersistent subjects contributes to improved viral control.

**Figure 2:**
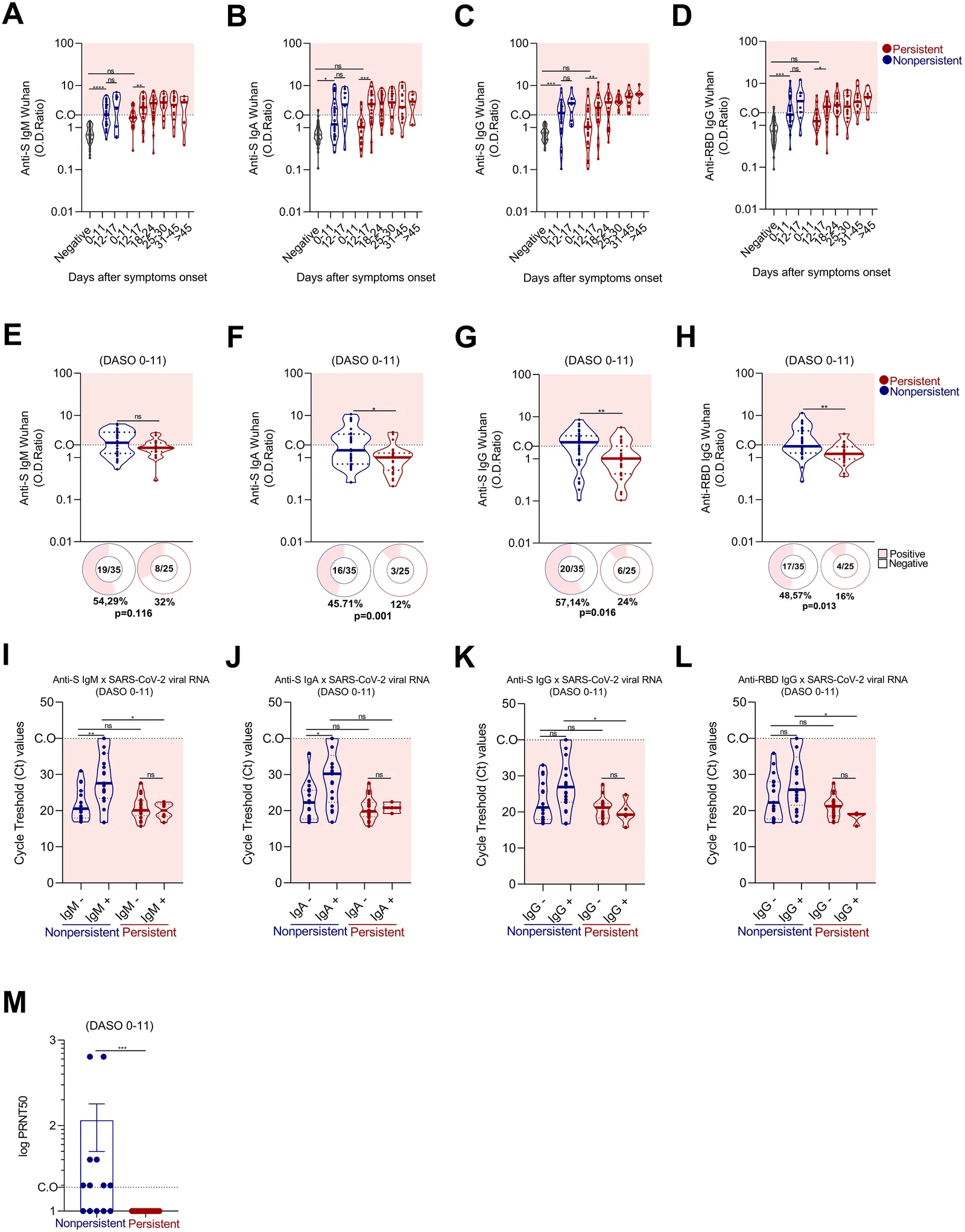
Viral persistence is associated with distinct early antibody responses to SARS-CoV-2. **(A–D)** Relative levels (OD ratio) of SARS-CoV-2-specific antibodies (anti-Spike IgM, IgA, and IgG, and anti-RBD IgG) in individuals infected with the ancestral Wuhan strain. Participants were stratified into persistent (red) and nonpersistent (blue) groups according to viral RNA persistence during the PCR-positive phase, based on days after symptom onset (DASO). PCR-negative controls are shown in gray. Relative antibody levels were calculated as the ratio between sample absorbance and the cutoff value (CO), defined as the mean absorbance of negative controls plus one standard deviation for each ELISA plate. Values above the cutoff were considered positive. **(E–H)** Donut charts indicating the proportion and percentage of seropositive and seronegative individuals in the persistent (n = 25) and nonpersistent (n = 35) groups during the early infection phase (0–11 DASO). **(I–L)** Association between antibody seroconversion and viral load, represented by PCR cycle threshold (Ct) values. Samples collected between 0 and 11 DASO were stratified according to the presence (Ig^+^) or absence (Ig^-^) of anti-Spike or anti-RBD antibodies. **(M)** Serum neutralizing activity against the ancestral Wuhan strain measured by PRNT_50_ in nonpersistent (blue, n = 13) and persistent (red, n = 14) individuals during early infection (0–11 DASO). Statistical analyses were performed using a mixed-effects model (REML) followed by Tukey’s multiple comparisons test or the Kruskal–Wallis test followed by Dunn’s multiple comparisons post-test, as appropriate. Comparisons between independent groups were performed using the Mann–Whitney U test, and differences in categorical variables, including seropositivity rates, were assessed using Fisher’s exact test. Statistical significance is indicated as follows: ns, not significant; *p < 0.05; **p < 0.01; ***p < 0.001; ****p < 0.0001.

### Early functional maturation and neutralization capacity predict viral clearance

Given the differences in seroconversion kinetics, we next evaluated whether qualitative features of the antibody response differed between groups during early infection. Using an indirect estimate of antibody affinity based on ACE2-blocking activity, measured by a multiplex competition assay performed in a subset of individuals, nonpersistent individuals exhibited higher inferred antibody affinity during the early phase compared with persistent individuals (**Supplementary Figure 1F**). Consistently, sera from nonpersistent subjects demonstrated greater neutralizing activity in PRNT assays against the ancestral Wuhan strain (**Figure 2M**). These findings indicate that not only the timing but also the quality of the early humoral response distinguishes nonpersistent from persistent individuals. Thus, the early generation of high-affinity, neutralizing antibodies likely contributes to rapid viral clearance.

### Humoral coordination and IgG subclass dynamics differ between persistent and nonpersistent individuals

Protein antigens typically induce T cell–dependent antibody responses dominated by IgG1 and IgG3 subclasses (17). Consistent with this pattern, the anti-Spike response in both groups was primarily mediated by IgG1. However, IgG1 appeared earlier in nonpersistent individuals (12–17 DASO) and later in persistent individuals (18–24 DASO) (**Figure 3A**). IgG3 was detected predominantly in persistent individuals after 18–24 DASO, whereas IgG2 and IgG4 remained largely undetectable in both groups across time (**Figures 3B–D**).

**Figure 3:**
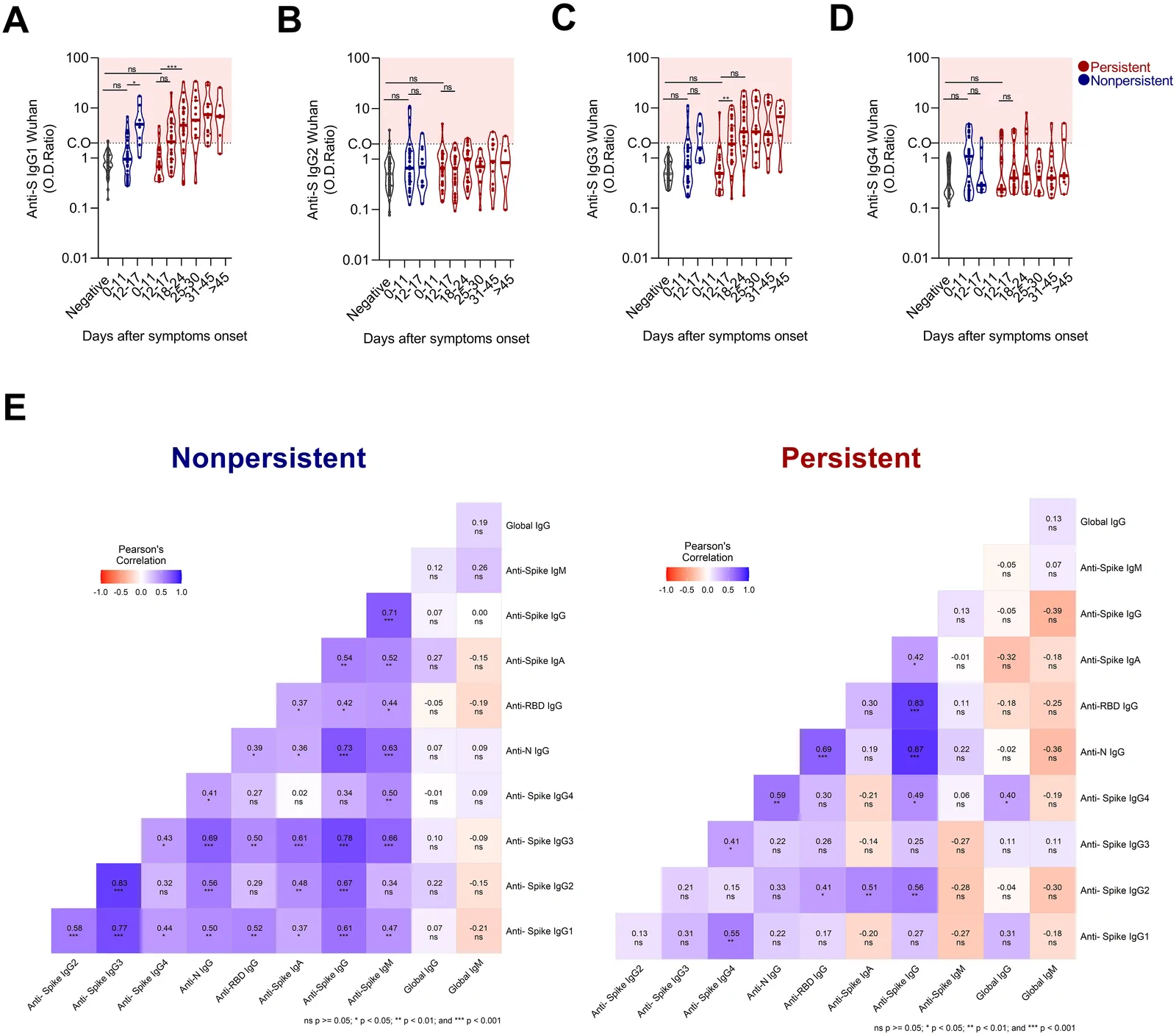
Humoral coordination and IgG subclass dynamics differ between persistent and nonpersistent infection. **(A–D)** Longitudinal kinetics of SARS-CoV-2 antigen-specific antibody responses in individuals previously infected with the Wuhan strain. The panels display the levels of anti-Spike IgG subclasses (IgG1–4) measured at various time points after symptom onset. Participants are stratified into nonpersistent (blue) and persistent (red) groups based on DASO classification, with PCR-negative controls shown in gray. Repeated-measures analyses were performed using a mixed-effects model (REML) followed by Tukey’s multiple comparisons test or the Kruskal–Wallis test followed by Dunn’s multiple comparisons post-test, as appropriate. **(E)** Correlation matrices displaying Pearson correlation coefficients between total immunoglobulins and antigen-specific antibodies (anti-Spike IgG1–4 subclasses, anti-Spike IgA/IgM/IgG, anti-RBD IgG, anti-N IgG, and total IgG/IgM) in nonpersistent (left, n = 32) and persistent (right, n = 32) individuals. Color intensity and hue indicate the strength and direction of the correlations (red = negative; blue = positive). Pearson correlation coefficients (r) are shown. Statistical significance: ns (not significant, p ≥ 0.05); *(p < 0.05); **(p < 0.01); ***(p < 0.001); ****(p < 0.0001).

To assess the coordination of the humoral response, correlation analyses were performed among antigen-specific antibodies and IgG subclasses. Nonpersistent individuals exhibited a highly integrated antibody profile, with strong positive correlations among anti-Spike IgG subclasses (IgG2-IgG3, r = 0.83; IgG1-IgG3, r = 0.77; IgG1-IgG2, r = 0.58; all p<0.001), as well as consistent correlations between anti-Spike, anti-RBD, and anti-N IgG responses. In contrast, persistent individuals showed a less coordinated correlation structure, with fewer and weaker associations among IgG subclasses and antigen-specific responses, suggesting reduced synchronization in IgG subclass maturation (**Figure 3E**). A non-significant trend toward inverse correlations between total IgM and IgG responses was observed in persistent individuals, a pattern not evident in nonpersistent subjects (**Figure 3E**). Although these associations did not reach statistical significance, they are consistent with a less coordinated humoral profile during early infection. These results indicate that coordinated maturation of IgG subclass responses is a feature of effective early humoral immunity underlying viral clearance.

### Primary infection induces durable but subclass-restricted humoral immunity

To determine whether viral persistence influenced long-term immunity, we evaluated antibody responses during the peri-infection phase (first PCR-negative sample) and late convalescence (>70 DASO). Anti-Spike IgM declined rapidly after viral clearance and became undetectable during late convalescence (**Figure 4A**). Anti-Spike IgA persisted longer but remained less prominent than IgG responses (**Figures 4B–D**). Both groups maintained robust anti-Spike and anti-RBD IgG responses during late follow-up, indicating durable humoral immunity independent of persistence status (**Figures 4C, D**).

**Figure 4:**
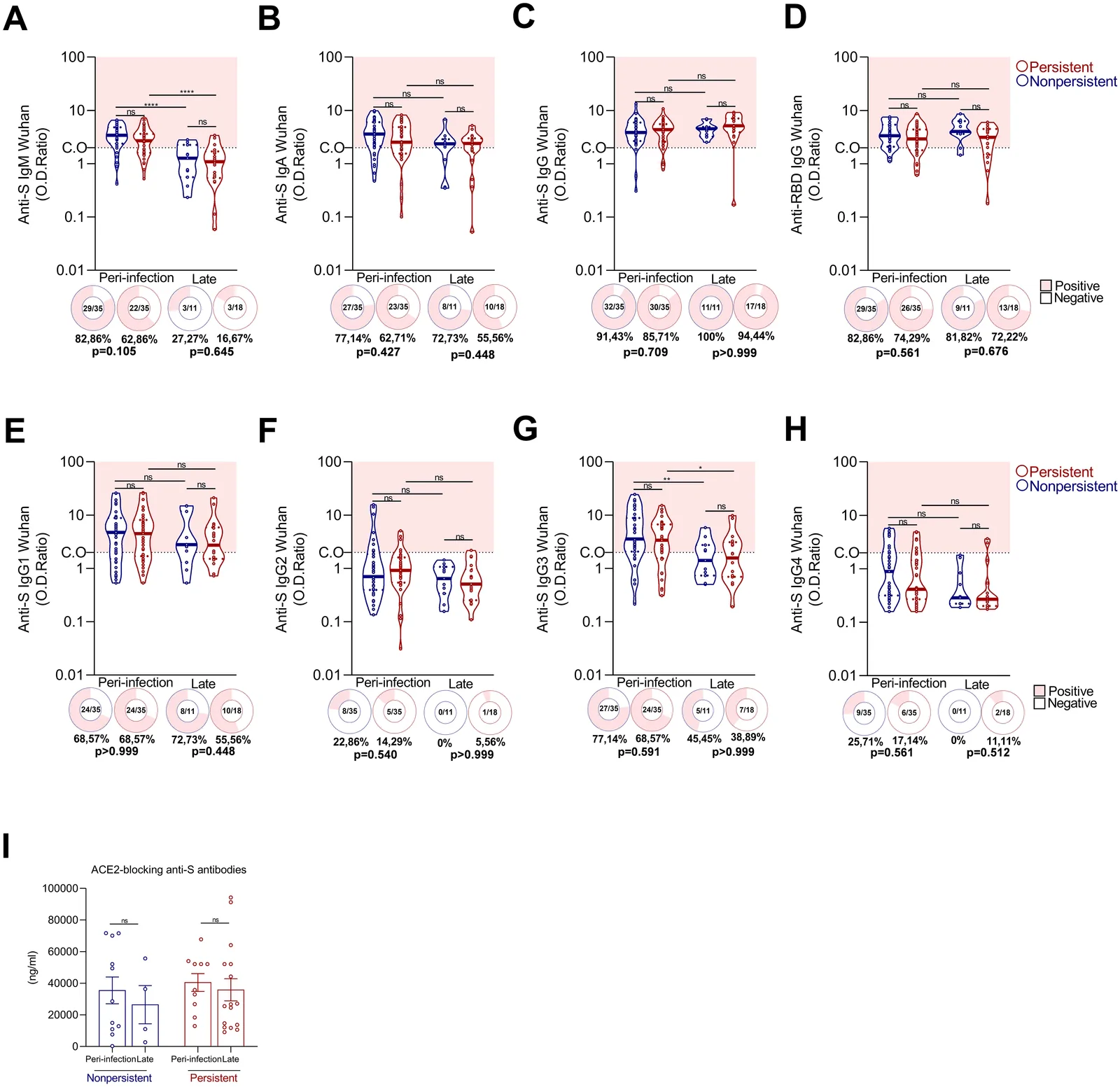
Sustained anti-Spike IgG and ACE2-blocking antibodies despite viral RNA persistence. **(A–D)** Relative levels (OD ratio) of anti-Spike (anti-S) IgM, IgA, IgG, and anti-RBD IgG in nonpersistent (blue) and persistent (red) individuals during the peri-infection and late convalescence phases. **(E–H)** Distribution and seroconversion rates of anti-S IgG subclasses (IgG1, IgG2, IgG3, and IgG4) across the same clinical groups and time points. Relative antibody levels were calculated as the ratio between sample absorbance and the cutoff value (CO), defined as the mean absorbance of negative controls plus one standard deviation for each ELISA plate. Values above the cutoff were considered positive. Donut charts indicate the number and percentage of seropositive individuals, with corresponding p-values for inter-group comparisons. **(I)** Concentration (ng/mL) of ACE2-blocking anti-Spike antibodies measured using multiplex competitive assay in nonpersistent and persistent individuals during the peri-infection and late convalescence phases. Statistical analyses were performed using the Kruskal–Wallis test followed by Dunn’s multiple comparisons post-test and the Mann–Whitney U test, as appropriate. Differences in categorical variables, including seropositivity rates, were assessed using Fisher’s exact test. Statistical significance is indicated as follows: ns, not significant; *p < 0.05; **p < 0.01; ****p<0.0001.

In both groups, IgG1 remained the predominant and most persistent subclass (**Figure 4E**), whereas IgG2 and IgG4 showed minimal seroconversion (**Figures 4F–H**). IgG3 anti-S declined over time (**Figure 4G**). Anti-N IgG levels decreased from the peri-infection to the late phase in the nonpersistent individuals (*p* < 0.01), whereas a similar trend did not reach statistical significance in the persistent group. However, inter-group anti-N IgG levels remained comparable at both time points (**Supplementary Figure 2A**). Importantly, high-affinity anti-Spike antibodies were maintained in both groups during late convalescence (**Figure 4I**). Individuals with persistent infection exhibited significantly higher total IgM levels during the peri-infection and late phase compared to nonpersistent subjects, consistent with extended antigen exposure during prolonged RNA detection (**Supplementary Figure 2B**). Nevertheless, total IgG levels remained stable across both phases (**Supplementary Figure 2C**). These results suggest that durable humoral immunity is established independently of viral persistence, despite differences in early infection dynamics.

### Early humoral responses are strain-focused and show limited cross-variant recognition

Throughout the COVID-19 pandemic, the emergence of SARS-CoV-2 variants raised the question of whether antibodies elicited by primary infection with the ancestral Wuhan strain could confer cross-reactivity. To address this, we evaluated anti-Spike IgG binding to Spike proteins from major variants of concern (Gamma, Beta, Delta, and Omicron). During the PCR-positive phase, binding to variant Spike proteins was generally low in both persistent and nonpersistent individuals (**Figures 5A–D**). Although isolated statistical differences were observed at specific time points, no consistent pattern of enhanced cross-variant recognition was detected in either group.

**Figure 5:**
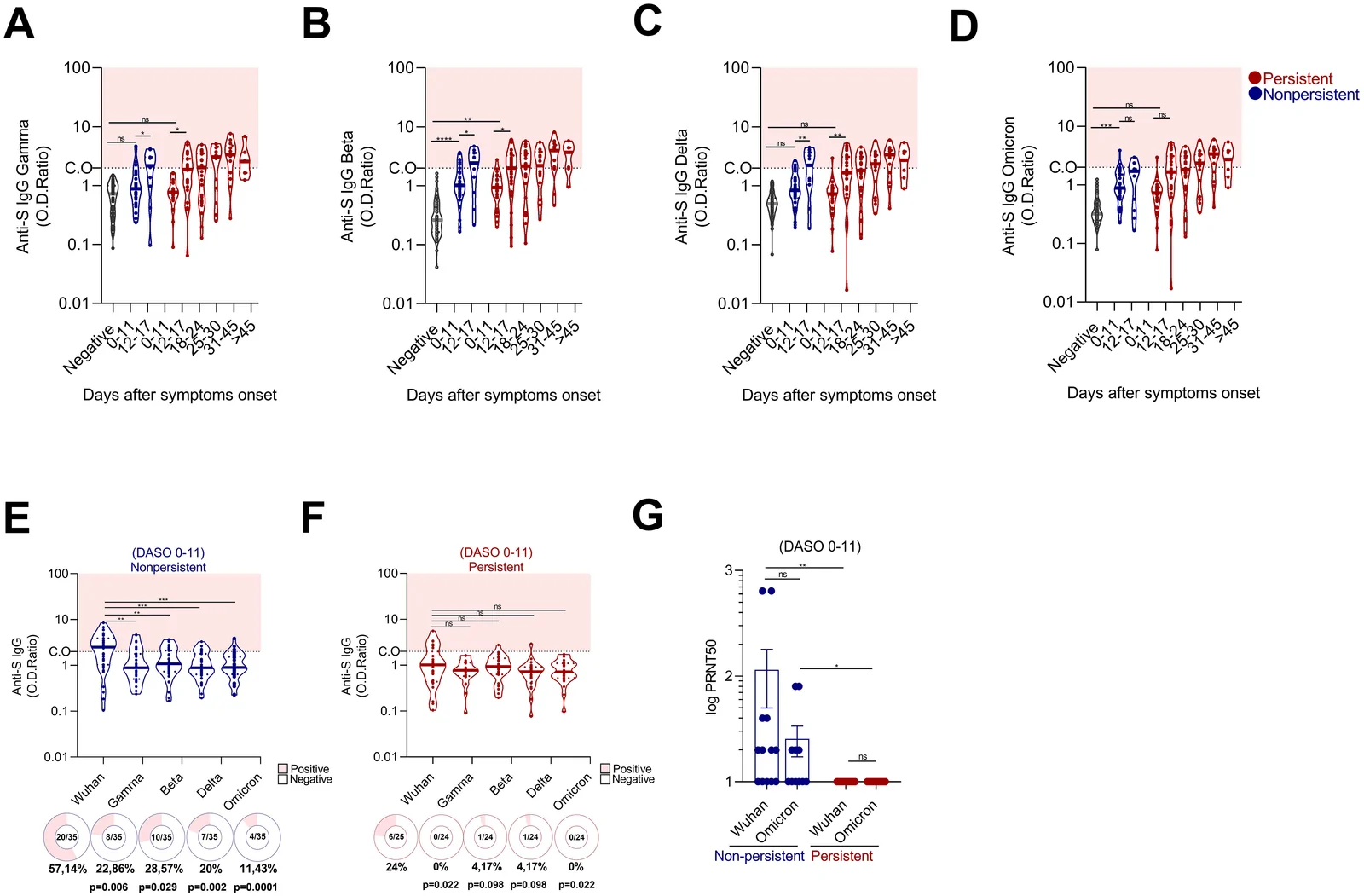
Dynamics of cross-reactive antibodies and early neutralizing capacity against SARS-CoV-2 variants. **(A–D)** Longitudinal kinetics of cross-reactive anti-Spike IgG responses against SARS-CoV-2 variants (Gamma, Beta, Delta, and Omicron) during the PCR-positive phase, stratified by days after symptom onset (DASO). PCR-negative controls are shown in gray. Relative antibody levels are expressed as OD ratios (sample absorbance/cutoff [CO]); values above the cutoff were considered positive. **(E, F)** Early cross-reactive anti-Spike IgG responses (0–11 DASO) against the ancestral Wuhan strain and variants of concern in nonpersistent (blue, n = 35) and persistent (red, n = 25) individuals. Donut charts indicate the number and percentage of seropositive individuals for each variant, with corresponding p-values for comparisons relative to the ancestral strain. **(G)** Early serum neutralizing antibody activity (0–11 DASO) against the Wuhan strain and Omicron variant in nonpersistent (n = 13) and persistent (n = 12) individuals, expressed as PRNT. Repeated-measures analyses were performed using a mixed-effects model (REML) followed by Tukey’s multiple comparisons test or the Kruskal–Wallis test followed by Dunn’s multiple comparisons post-test, as appropriate. Comparisons between independent groups were performed using the Mann–Whitney U test, while paired comparisons were analyzed using the Wilcoxon matched-pairs signed-rank test. Differences in categorical variables, including seropositivity rates, were assessed using Fisher’s exact test. Statistical significance is indicated as follows: ns, not significant; *p<0.05; **p<0.01; ***p<0.001; ****p<0.0001.

We next focused on the early phase (0–11 DASO), a critical window for viral clearance. Individuals with nonpersistent infection mounted a robust IgG response against the ancestral Spike during this period. However, binding to variant Spike proteins was significantly reduced compared with the ancestral strain, indicating a predominantly strain-specific response (**Figure 5E**). In contrast, persistent individuals exhibited weaker early recognition of the ancestral antigen and minimal reactivity to variant Spike proteins (**Figure 5F**). Analysis of anti-RBD IgG responses yielded a similar pattern. In nonpersistent individuals, early binding was strongest to the ancestral RBD and declined markedly for Gamma, Delta, and Omicron (**Supplementary Figure 3A**). Persistent individuals showed low overall reactivity, with limited evidence of variant recognition during this early period (**Supplementary Figure 3B**). Direct comparisons between groups confirmed that cross-variant IgG binding did not significantly differ between persistent and nonpersistent individuals during early infection (**Supplementary Figure 3C, D**). Consistently, neutralizing activity during this early phase was largely restricted to nonpersistent individuals, who exhibited detectable titers predominantly against the ancestral Wuhan strain, with limited cross-neutralization against Omicron (**Figure 5G**). In contrast, neutralizing activity in persistent individuals was minimal or undetectable. Together, these findings indicate that early viral clearance is associated with accelerated functional maturation of the antibody response, characterized by the rapid generation of antibodies directed to the ancestral strain, rather than with broad cross-variant humoral recognition.

### Cross-variant seroreactivity during convalescence remains limited and does not significantly differ between groups

We next examined whether cross-variant recognition evolved after viral clearance. During the peri-infection phase (first PCR-negative sample), both persistent and nonpersistent individuals displayed robust anti-Spike IgG responses against the ancestral strain (**Figures 6A, B**). Binding to variant Spikes was detectable in a subset of individuals in both groups. Persistent individuals showed numerically higher proportions of seroreactivity for some variants during this phase. However, direct comparisons between groups did not reveal statistically significant differences (**Figure 6C**). Analysis of anti-RBD IgG responses during peri-infection phase revealed a more pronounced reduction in variant recognition compared to full Spike, particularly for Omicron (**Supplementary Figures 4A, B**). Overall, cross-variant recognition remained limited and did not significantly differ between groups (**Supplementary Figure 4C**).

**Figure 6:**
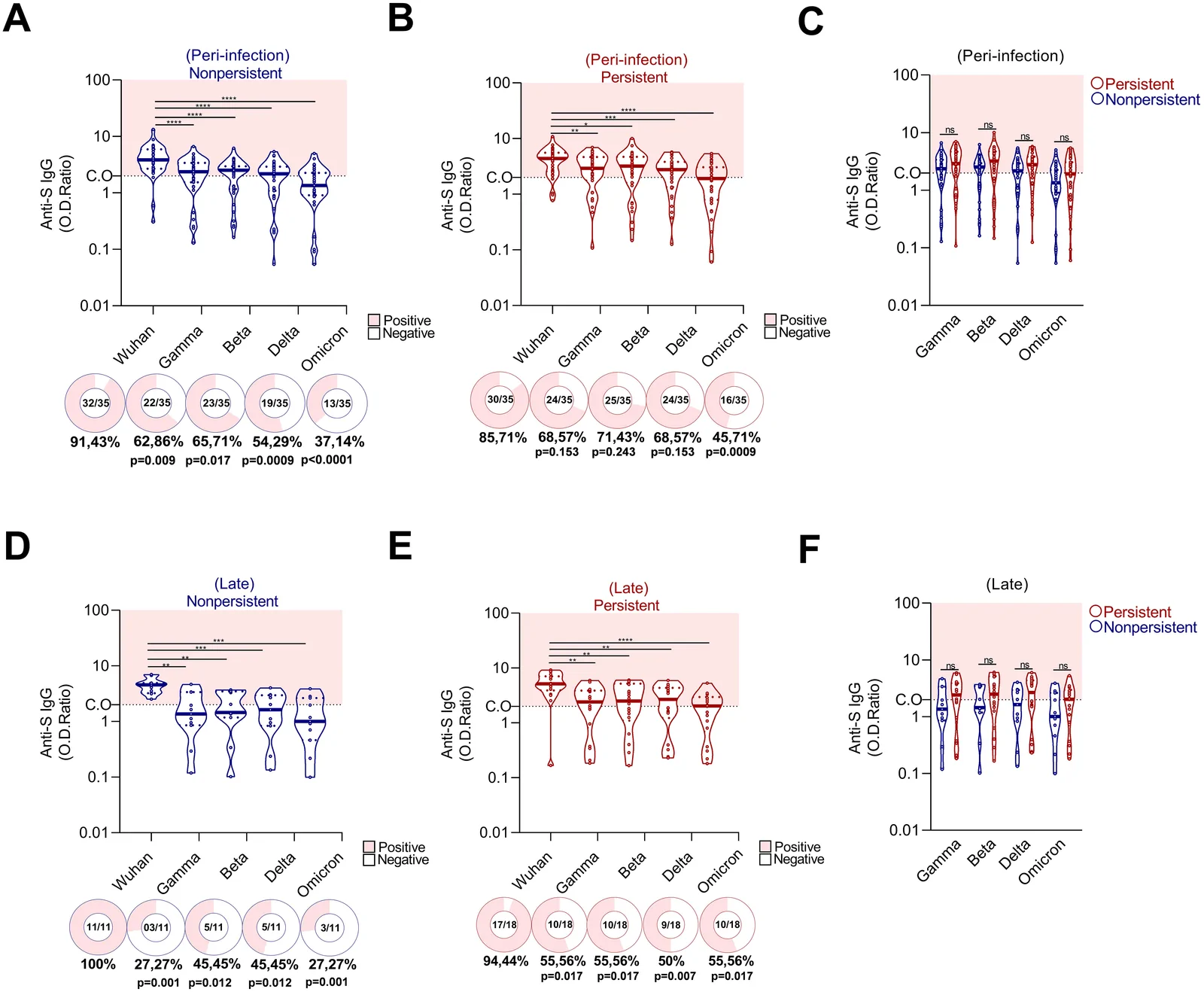
Cross-reactive anti-Spike IgG responses to SARS-CoV-2 variants during peri-infection and late convalescence. **(A, B, D, E)** Relative levels (OD ratio) of cross-reactive anti-Spike IgG against the ancestral Wuhan strain and SARS-CoV-2 variants (Gamma, Beta, Delta, and Omicron) in nonpersistent (blue) and persistent (red) individuals during the peri-infection **(A, B)** and late convalescence **(D, E)** phases. **(C, F)** Inter-group comparisons of antibody levels during the peri-infection and late convalescence phases, respectively. Donut charts indicate the proportion of seropositive individuals, with corresponding p-values for group comparisons. Repeated-measures analyses were performed using a mixed-effects model (REML) followed by Tukey’s multiple comparisons test. Comparisons between independent groups were performed using the Mann–Whitney U test. Differences in categorical variables, including seropositivity rates, were assessed using Fisher’s exact test. Statistical significance is indicated as follows: ns, not significant; *p < 0.05; **p < 0.01; ***p < 0.001; ****p < 0.0001.

During the late convalescence phase, both groups exhibited a reduction in cross-reactive anti-Spike IgG antibodies against variants relative to the ancestral Wuhan strain. However, the persistent group maintained a broader recognition profile, with approximately 50-55% of individuals still recognizing the variants, including the highly divergent Omicron strain (55.56% seropositivity) (**Figure 6E**). In contrast, the nonpersistent group demonstrated a more drastic reduction in cross-reactivity as the genetic distance from the ancestral strain increased, with seropositivity dropping to 27.27% against Omicron (**Figure 6D**). Notably, despite these distinct intra-group seroprevalence trends, direct quantitative comparisons of cross-reactive antibody levels between persistent and nonpersistent individuals revealed no statistically significant differences for any of the tested variants (**Figure 6F**). Analysis of anti-RBD IgG responses during late follow-up showed similarly reduced cross-variant recognition without significant differences between groups (**Supplementary Figures 4D–F**).

Collectively, these data demonstrate that primary infection with the ancestral SARS-CoV-2 strain induces durable strain-specific humoral immunity. Cross-variant seroreactivity persists in a subset of individuals but does not significantly differ between persistent and nonpersistent groups during follow-up.

### Vaccination sustains and amplifies anti-Spike humoral responses independently of prior viral persistence

We next asked whether prior viral persistence influenced the magnitude or quality of vaccine-induced humoral responses. To address this, we longitudinally analyzed antibody levels before and after vaccination, grouping samples according to days since the start of immunization. Participants received predominantly heterologous vaccine regimens, including CoronaVac, AstraZeneca, Pfizer, and booster doses with mRNA or adenoviral platforms. Following infection resolution, anti-Spike IgM levels declined during convalescence and remained low after vaccination, with occasional increases compatible with possible reinfection events (**Figures 4A**, **7A**). In contrast, vaccination sustained and progressively increased anti-Spike IgA and IgG levels over time in both persistent and nonpersistent individuals (**Figures 7B, C**). Anti-RBD IgG responses followed a similar pattern (**Figure 7D**). No consistent differences between groups were observed at any evaluated time point.

**Figure 7:**
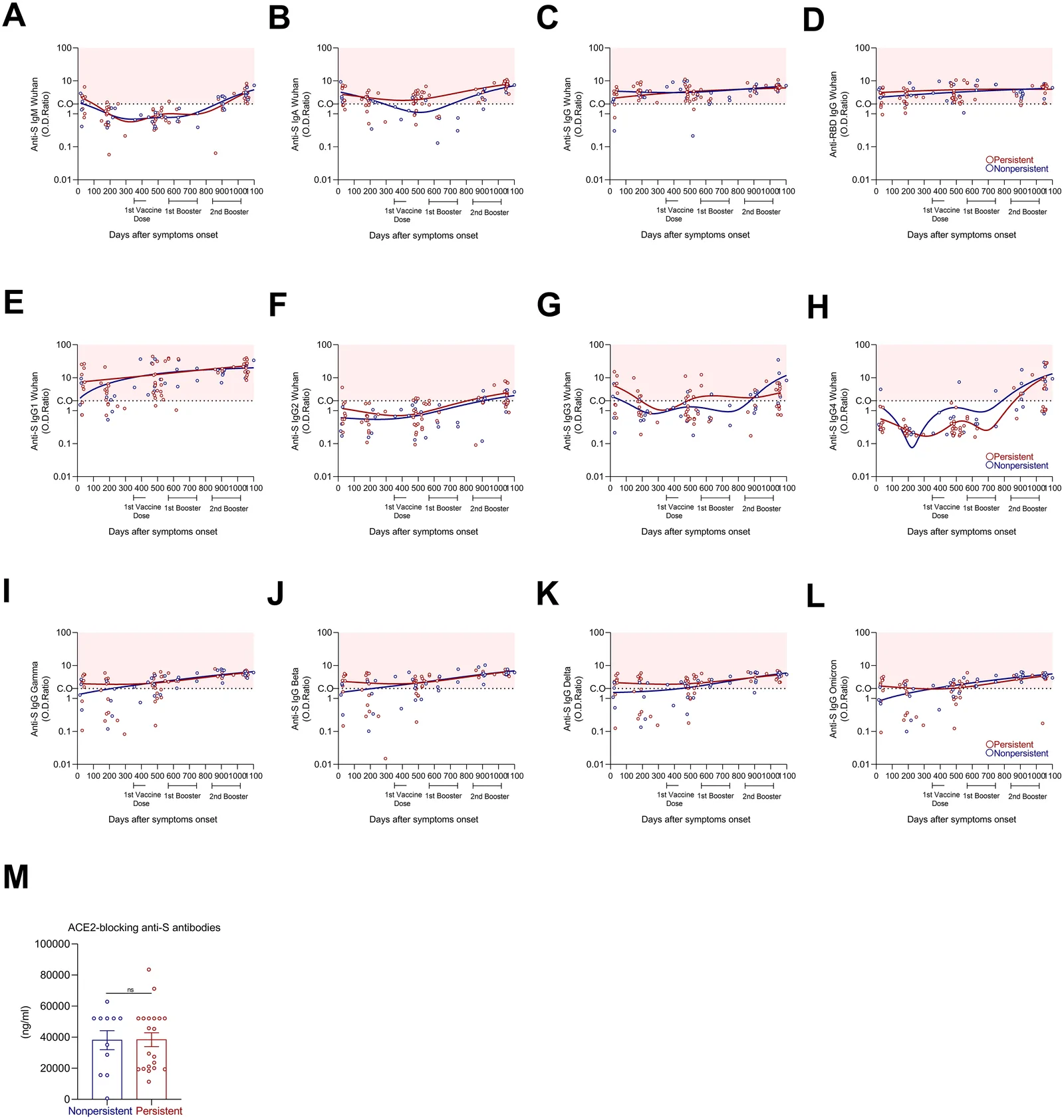
Longitudinal dynamics of SARS-CoV-2-specific antibody responses following vaccination in convalescent individuals. **(A–L)** Longitudinal dynamics of anti-Spike antibody responses following vaccination in persistent (red, n = 23) and nonpersistent (blue, n = 10) individuals. **(A–C)** show anti-Spike IgM, IgA, and IgG responses against the ancestral Wuhan strain, respectively, while **(D)** shows anti-RBD IgG levels. **(E–H)** represent anti-Spike IgG subclass responses (IgG1–IgG4) against the Wuhan strain. **(I–L)** show cross-reactive anti-Spike IgG responses against SARS-CoV-2 variants of concern (Gamma, Beta, Delta, and Omicron). Antibody kinetics were monitored longitudinally from the last convalescence time point through the post-vaccination period. Relative antibody levels are expressed as OD ratios (sample absorbance/cutoff [CO]); values above the cutoff were considered seropositive. Solid lines represent Lowess regression curves. **(M)** Concentration (ng/mL) of ACE2-blocking anti-Spike antibodies measured using multiplex competitive assay, between nonpersistent (n = 10) and persistent (n = 20) individuals following vaccination. Statistical analyses were performed using the Mann–Whitney U test. ns, not significant.

In both groups, analysis of IgG subclasses showed that IgG1 remained the predominant subclass after vaccination (**Figure 7E**). Low levels of IgG2, IgG3, and IgG4 were observed, with modest increases following repeated antigen exposure being most apparent for IgG3 and IgG4 (**Figures 7F–H**). However, subclass kinetics were broadly comparable between groups. Vaccination also enhanced IgG binding to variant Spike proteins in both groups, including Gamma, Beta, Delta, and Omicron (**Figures 7I–L**). Variant recognition increased progressively following primary vaccination and booster doses, with similar trajectories in persistent and nonpersistent individuals. Finally, titers of high-affinity anti-Spike antibodies measured after vaccination did not significantly differ between groups (**Figure 7M**).

Collectively, these data indicate that vaccination robustly amplifies and sustains anti-Spike humoral responses in previously infected individuals, independently of prior viral persistence status.

## Discussion

The determinants of viral clearance during acute SARS-CoV-2 infection remain incompletely defined, particularly in individuals with mild disease. In our previous longitudinal analysis of this cohort, we identified altered innate immune signatures and described differences in anti-Spike binding responses associated with prolonged viral RNA detection (25). However, the functional implications of these humoral differences and their relationship to viral clearance kinetics remained unresolved. In this longitudinal study spanning three years, we investigated the relationship between humoral immune dynamics and prolonged viral RNA in mild-to-moderate COVID-19. Our findings reveal that early coordination and functional quality of the antibody response dictate initial viral clearance (**Figures 1**, **2**). Consistently, viral RNA clearance is associated with the early generation of high-affinity, neutralizing anti-Spike antibodies, whereas total immunoglobulin levels do not distinguish nonpersistent from persistent infection (**Figure 2**; **Supplementary Figure 1**). Together, these findings indicate that the timing and functional maturation of the antibody response, rather than its magnitude alone, are key determinants of efficient viral control. Importantly, this early divergence does not compromise the establishment of durable immune memory or the subsequent responsiveness to vaccination in individuals with prolonged viral RNA shedding.

The critical window for establishing viral control occurred within the first 11 days after symptom onset, during which nonpersistent individuals mounted accelerated seroconversion to Spike and RBD. This early response was accompanied by higher inferred antibody affinity and greater neutralizing activity directed against the ancestral strain, corroborating previous reports that prompt and robust antibody generation is a hallmark of “quick healers” and early viral controllers (31, 32). Notably, persistent individuals exhibited a greater viral RNA burden during this early phase, as reflected by significantly lower Ct values, despite showing delayed seroconversion and reduced early functional antibody responses. This observation indicates that greater early viral RNA burden alone is insufficient to drive a more efficient humoral response. Early signals of affinity maturation, even when assessed in a subset of individuals, were consistent with functional neutralization, reinforcing the link between antibody quality and viral control (**Figure 2**; **Supplementary Figure 1**).

Building on our previous work (25), which identified altered innate immune signatures and reported increased antibody binding capacity in individuals with prolonged infection, the present study provides a temporal and functional refinement of these observations. While the previous study primarily focused on early innate responses and antibody features emerging at later time points, it did not resolve the kinetics of early seroconversion. Here, by specifically interrogating the 0–11 DASO window, we demonstrate that accelerated seroconversion and early acquisition of functional antibody activity are key determinants of viral clearance, rather than late increases in antibody binding capacity (**Figure 2**; **Supplementary Figure 1**). Notably, although persistent individuals were previously shown to develop antibodies with enhanced binding capacity at later stages, our data indicate that this delayed maturation does not compensate for the lack of early functional responses required for efficient viral control.

High-affinity antibody production is classically linked to germinal center–dependent maturation (20, 21), and longitudinal analyses of SARS-CoV-2 infection have demonstrated continued antibody evolution over time (13, 14). Although we did not directly assess germinal center activity or T follicular helper cell dynamics, the serological patterns observed here are consistent with more rapid functional maturation of the humoral response in individuals who efficiently cleared viral RNA.

In contrast, persistent individuals exhibited delayed seroconversion, prolonged IgM responses, and reduced coordination among IgG subclasses during early infection. These features suggest differences in the timing of class switching and maturation dynamics (**Figures 2**, **3**). Previous longitudinal analyses have demonstrated that severe COVID-19 is characterized by “immunological misfiring, “ in which delayed and uncoordinated innate and adaptive immune responses fail to rapidly clear the virus, allowing sustained viral loads that drive clinical deterioration and disease severity (33). Further reinforcing the critical nature of this timeline, the delayed production of neutralizing antibodies, particularly beyond 14 days post-symptom onset, has been directly correlated with impaired viral control and fatal outcomes in cohorts with severe cases (34). Furthermore, severe COVID-19 has been associated with dysregulated extrafollicular activation and disrupted germinal center formation (35, 36). Although our cohort consisted exclusively of mild-to-moderate cases, the less synchronized humoral profile and delayed kinetics observed in persistent individuals mirror this concept of immune dyscoordination on a milder scale, associated with prolonged viral RNA shedding rather than severe clinical outcomes. Our findings, therefore, extend the concept that even subtle variations in early humoral maturation kinetics, within clinically mild disease, may influence viral persistence. The association between early neutralizing capacity and clearance supports a model in which prompt functional antibody generation limits ongoing viral replication before prolonged antigenic exposure occurs.

Importantly, viral clearance in this cohort reflects immune responses to the ancestral Wuhan strain, and differences in cross-variant recognition were not associated with viral clearance. During acute infection, antibody responses were largely strain-focused toward the ancestral Wuhan Spike in both groups, and cross-variant neutralization was limited, consistent with previous reports of narrow primary neutralizing repertoires (22, 23). Clearance was associated with the early emergence of functional antibodies directed against the infecting strain rather than with broader cross-reactivity to divergent variants.

Despite the delayed acute response in the persistent group, our long-term follow-up revealed a convergence in humoral memory. Early in the pandemic, the durability of humoral immunity following mild COVID-19 was a subject of intense debate, with initial studies reporting a rapid decay of circulating anti-SARS-CoV-2 antibodies within the first few months (37, 38). However, subsequent long-term evaluations demonstrated that after an initial contraction phase, robust neutralizing antibody and IgG responses stabilize and persist in serum and saliva for several months to years (12, 39–41). In agreement with these latter findings, during convalescence, both persistent and nonpersistent individuals maintained durable anti-Spike and anti-RBD IgG responses, predominantly of the IgG1 subclass (**Figure 4**). IgG1 and IgG3 are hallmarks of Th1-biased antiviral immunity (17), and the progressive decline of IgG3 is consistent with its shorter half-life. Persistent individuals exhibited higher total IgM levels during the peri-infection phase, consistent with extended antigen exposure due to prolonged viral RNA shedding (**Supplementary Figure 2**). However, this prolonged exposure does not seem to confer a measurable advantage in cross-variant antibody breadth (**Figure 5**; **Supplementary Figure 3**). Although persistent individuals exhibited a numerical trend toward broader cross-variant seroreactivity during later time points, these differences were not statistically significant and did not translate into enhanced functional antibody breadth (**Figure 6**; **Supplementary Figure 4**).

These findings indicate that primary infection with the ancestral strain predominantly imprints a strain-focused humoral memory, and that prolonged viral RNA detection alone is insufficient to drive broad cross-neutralization. While extended antigen exposure may contribute to subtle differences in humoral maturation dynamics, it does not translate into enhanced cross-variant antibody breadth during follow-up. In contrast, repeated antigen exposure through vaccination has been shown to promote the expansion and evolution of cross-reactive memory B cell clones, leading to increased neutralization breadth capable of overcoming major antigenic shifts, such as those presented by the Omicron variant (24, 42), highlighting that the quality and context of antigen exposure, rather than its duration alone, are critical determinants of humoral diversification. Together, these observations suggest that the kinetics and functional quality of adaptive immunity may be particularly important for early viral control, whereas the breadth and plasticity of the antibody repertoire become increasingly relevant for protection against antigenically divergent variants. In previously uninfected vaccinated individuals, pre-existing vaccine-induced immune memory may similarly accelerate adaptive recall upon subsequent infection, with the timing of recall potentially contributing to viral control, as observed in studies of breakthrough infection (43, 44).

Vaccination markedly reshaped the humoral landscape in both groups. Immunization with heterologous platforms robustly amplified anti-Spike IgA and IgG levels over time. While IgG1 remained the predominant subclass after vaccination, repeated antigen exposure through booster regimens drove specific subclass dynamics, including modest increases in IgG2, IgG3, and IgG4 (**Figure 7**). Consistent with recent reports demonstrating the emergence of IgG4 alongside sustained overall IgG effector functions following repeated mRNA vaccination (45), we observed this specific IgG4 elevation after booster doses. Importantly, the magnitude of this subclass shift is heavily influenced by vaccine platform-specific priming and immune imprinting; repeated mRNA vaccination typically drives a more pronounced IgG4 expansion than other platforms (46). This platform dependency likely explains why the IgG4 increases remained modest in our cohort, which received diverse heterologous regimens encompassing inactivated and adenoviral vector vaccines.

Beyond subclass dynamics, booster exposure progressively enhanced IgG binding to variant Spike proteins, including Gamma, Beta, Delta, and Omicron, and maintained high-affinity responses. Crucially, these vaccine-induced amplifications in titers, variant recognition, and antibody affinity occurred with similar trajectories in both persistent and nonpersistent individuals (**Figure 7**). Indeed, it has been clearly demonstrated that while primary SARS-CoV-2 infection in unvaccinated individuals elicits a narrowly focused, strain-specific humoral immunity, it is the integration of vaccination that critically broadens antibody cross-reactivity and enhances Fc effector functions against diverse variants of concern (47). Furthermore, our observation of progressive cross-variant recognition is in agreement with recent findings demonstrating that booster doses are essential for driving affinity maturation and generating robust cross-variant neutralization, particularly against highly divergent lineages like Omicron and its subvariants (48). These results align with studies showing that vaccination induces robust, cross-reactive IgG responses in previously infected individuals, effectively leveling the immunological playing field regardless of initial infection dynamics (49). Therefore, our data confirm that early differences in infection kinetics and prolonged viral RNA shedding do not constrain subsequent vaccine responsiveness.

This study has limitations. Viral persistence was defined by RT-qPCR detection of viral RNA and does not necessarily reflect replication-competent virus throughout the period of positivity (4). Moreover, our analysis was restricted to nasopharyngeal viral RNA detection, and extrapulmonary tissues were not available for assessment of persistent viral proteins. Therefore, we cannot exclude the possibility of tissue-associated viral persistence beyond the respiratory tract. Previous analyses from the same study population identified distinct innate immune features associated with prolonged SARS-CoV-2 infection (25). However, because long-term symptoms were not systematically assessed, the present study cannot determine whether prolonged viral RNA detection is associated with post-COVID-19 manifestations. We did not directly evaluate T cell responses, Tfh dynamics, or clonal evolution at the B cell receptor level, which would provide mechanistic confirmation of the maturation differences inferred from serological analyses. Furthermore, all participants were infected with the ancestral strain during the first epidemic wave, which corresponds to the phase in which viral persistence was defined; therefore, immune kinetics may differ in infections caused by later variants with enhanced immune-evasion properties. In addition, information on potential reinfections during the latter follow-up period was not systematically available, which limits our ability to assess their contribution to the evolution of cross-reactive humoral responses over time. The longitudinal design of the cohort inherently led to some loss to follow-up over the three-year period. The finite volume of serum collected at specific time points restricted our capacity to perform all downstream immunological assays (such as neutralization assays and ACE2-blocking assays) across the entire cohort. Consequently, the sample size (n) varies across different analyses. Nevertheless, despite this sample attrition, the remaining sample sizes were sufficient to identify robust and consistent immunological signatures distinguishing the persistent and nonpersistent phenotypes.

In summary, our data support a model in which the timing and functional quality of the early humoral response are decisive for SARS-CoV-2 RNA clearance, even in clinically mild infection. Rapid maturation and early neutralizing activity, rather than antibody magnitude or breadth, are associated with efficient viral control. Prolonged viral RNA reflects delayed humoral kinetics but does not necessarily translate into superior long-term variant breadth. Importantly, vaccination consistently elicited stronger and broader humoral responses in both groups, underscoring its critical role in shaping effective and cross-reactive immunity regardless of prior viral persistence status. Together, these findings emphasize that the tempo of antibody functional maturation is a critical parameter shaping both acute infection outcomes and the subsequent immune landscape, linking early humoral kinetics to long-term immune architecture.

## Data Availability

The original contributions presented in the study are included in the article/**Supplementary Material**. Further inquiries can be directed to the corresponding authors.

## In memoriam

We honor Dr. Amilcar Tanuri for his scientific contributions and dedication to this work.
