# Supplementary material for: Early functional maturation of anti-Spike antibodies predicts efficient SARS-CoV-2 RNA clearance
Source: Front Immunol. 2026 Oct 2;17:1948045. doi: 10.3389/fimmu.2026.1948045 (PMC13672549; doi:10.3389/fimmu.2026.1948045)
Supplement: Supplementary file 1 [file DataSheet1.pdf]

Supplementary Material

Early functional maturation of anti-Spike antibodies predicts efficient SARS-CoV-2 RNA clearance in mild infection

Danielle Rodrigues *et al.*

This file includes:

Figures S1 to S4

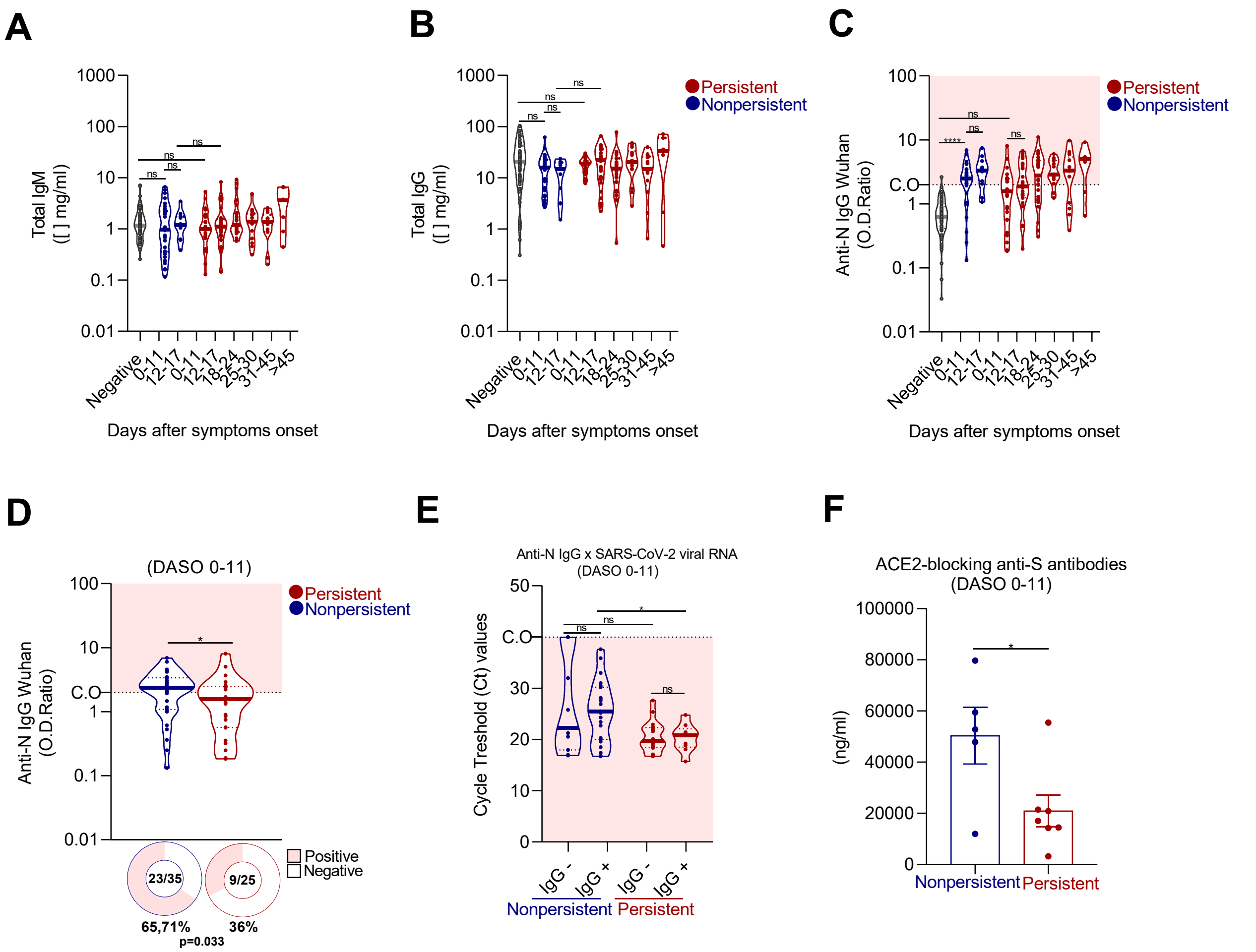

Supplementary Figure 1: Early humoral responses and ACE2-blocking activity in persistent and nonpersistent SARS-CoV-2 infection. (A–B) Serum concentrations of total IgM and total IgG antibodies in individuals infected with SARS-CoV-2. (C) Distribution of antigen-specific anti-N IgG responses in individuals previously infected with the ancestral Wuhan strain. Participants were stratified into persistent (red) and nonpersistent (blue) groups according to viral RNA persistence during the PCR-positive phase, based on days after symptom onset (DASO). PCR-negative controls are shown in gray. Relative antibody levels (OD ratio) were calculated by dividing the optical density by the cutoff value (CO). The cutoff was established as the mean absorbance of negative controls plus one standard deviation for each ELISA plate. OD ratios above the cutoff were considered positive, whereas values below the cutoff were considered negative. (D) Donut charts indicate the proportion of seropositive and seronegative individuals in each group during the early infection phase (0–11 DASO). Differences in categorical variables, including seropositivity rates, were assessed using Fisher's exact test. (E) Association between anti-N IgG seroconversion and viral load, represented by PCR cycle threshold (Ct) values. Samples collected between 0 and 11 DASO were stratified according to the presence (IgG^{+}) or absence (IgG^{-}) of anti-N antibodies. (F) Concentration (ng/mL) of ACE2-blocking anti-Spike antibodies measured using a multiplex competitive assay in a subset of persistent (n = 7) and nonpersistent (n = 5) individuals during early infection (0–11 DASO). Repeated-measures analyses were performed using a mixed-effects model (REML) followed by Tukey's multiple comparisons test

or the Kruskal–Wallis test followed by Dunn's multiple comparisons post-test, as appropriate. Comparisons between independent groups were performed using the Mann–Whitney U test. Statistical significance is indicated as follows: ns, not significant; *$p < 0.05$; ****$p < 0.0001$.

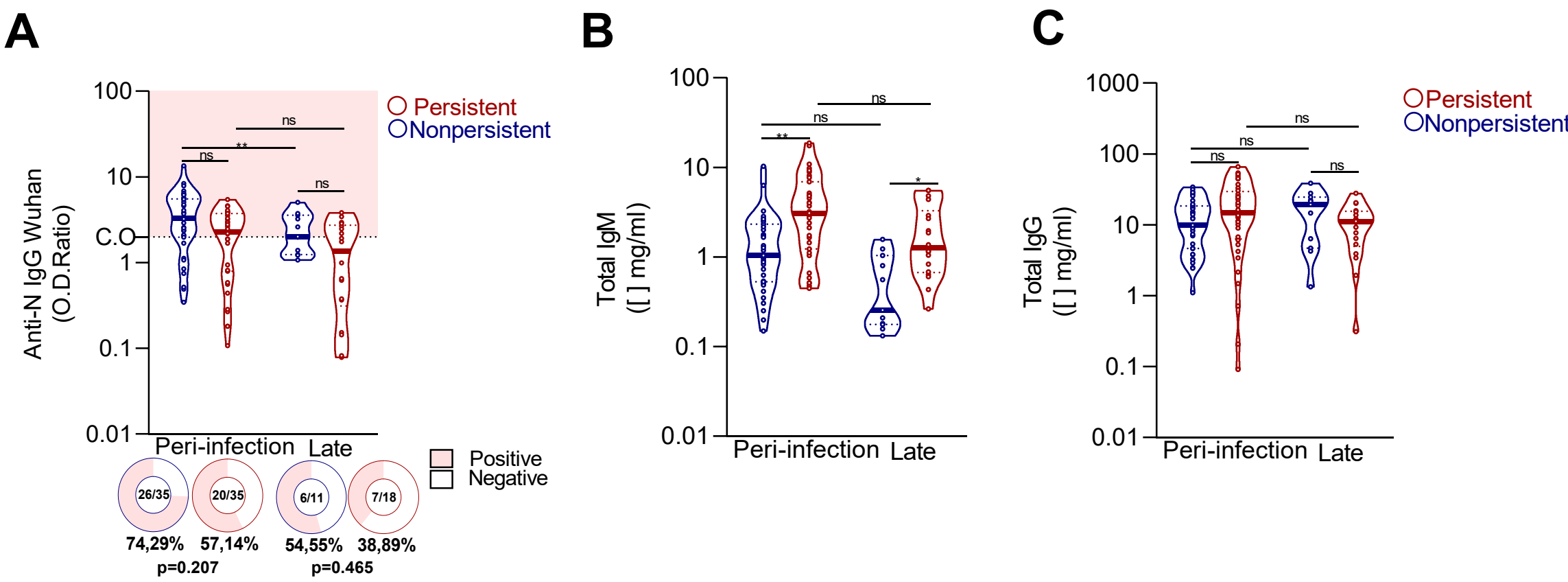

Supplementary Figure 2: Longitudinal dynamics of anti-Nucleocapsid IgG and total immunoglobulins during peri-infection and late convalescence. (A) Relative levels (OD ratio) of anti-Nucleocapsid (anti-N) IgG in nonpersistent (blue) and persistent (red) individuals during the peri-infection and late convalescence phases. Donut charts indicate the proportion of anti-N IgG-seropositive individuals in each group, with corresponding p-values for inter-group comparisons. Differences in categorical variables, including seropositivity rates, were assessed using Fisher's exact test. (B–C) Serum concentrations (mg/mL) of total IgM (B) and total IgG (C) in nonpersistent and persistent individuals during the peri-infection and late convalescence phases. Statistical analyses were performed using the Kruskal–Wallis test followed by Dunn's multiple comparisons post-test, and paired comparisons were performed using the Wilcoxon matched-pairs signed-rank test. Statistical significance is indicated as follows: ns, not significant; **p < 0.01.

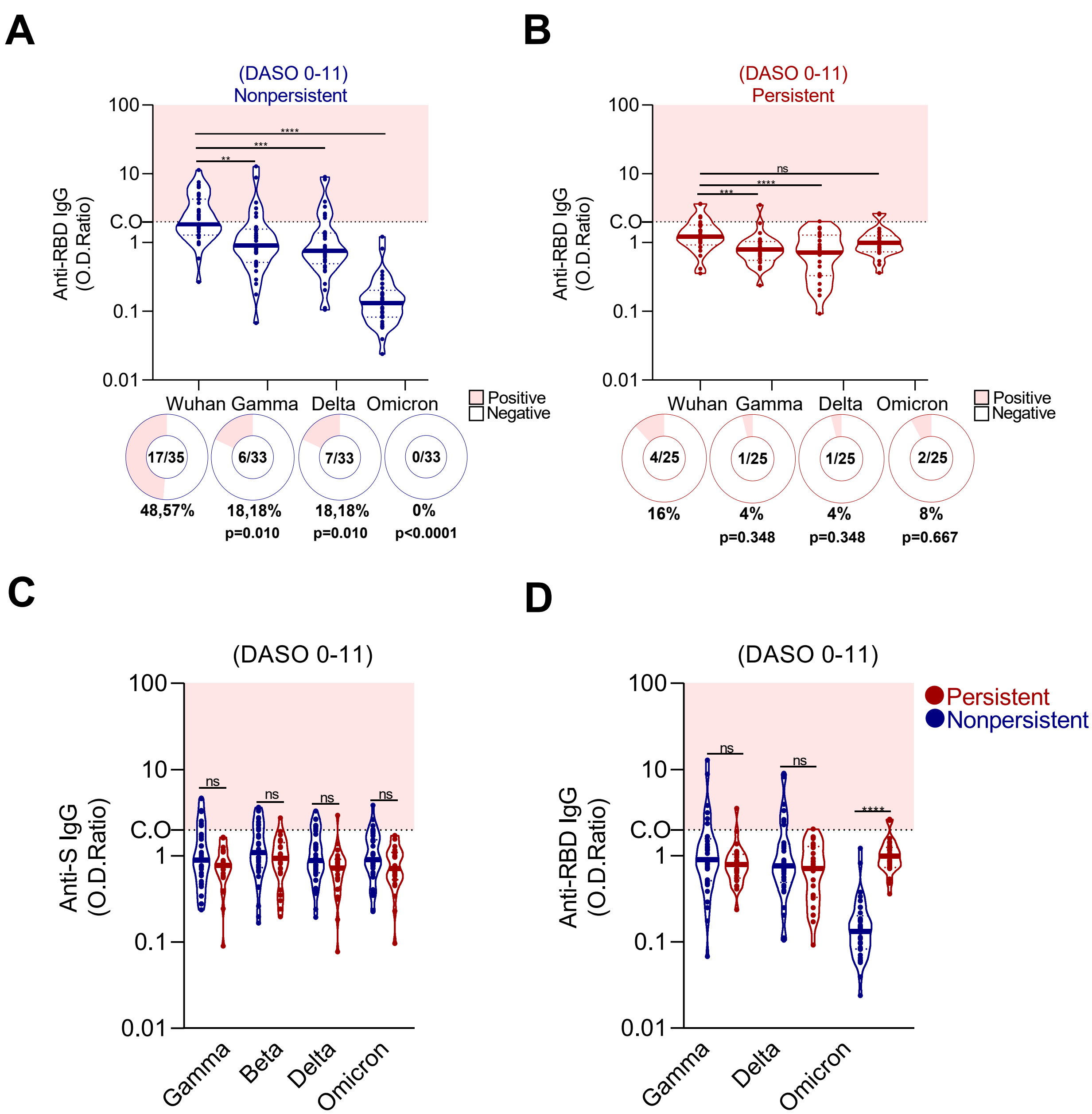

Supplementary Figure 3: Early cross-reactive anti-Spike and anti-RBD IgG responses against SARS-CoV-2 variants in persistent and nonpersistent infection. (A–B) Early cross-reactive anti-RBD IgG responses (0–11 DASO) against the ancestral Wuhan strain and SARS-CoV-2 variants of concern (Gamma, Delta, and Omicron) in nonpersistent (blue) and persistent (red) individuals. The horizontal dashed line (CO) indicates the seropositivity cutoff, and the pink shaded area represents the positive range. Donut charts indicate the proportion (n/N) and percentage of seropositive individuals, with corresponding p-values for comparisons relative to the ancestral Wuhan strain. (C–D) Inter-group comparisons of early anti-Spike and anti-RBD IgG levels between nonpersistent and persistent individuals across SARS-CoV-2 variants. Repeated-measures analyses were performed using a mixed-effects model (REML) followed by Tukey's multiple comparisons test or the Friedman test followed by Dunn's multiple comparisons test, as appropriate. Comparisons between independent groups were

performed using the Mann–Whitney U test. Differences in categorical variables, including seropositivity rates, were assessed using Fisher's exact test. Statistical significance is indicated as follows: ns, not significant; **$p < 0.01$; ***$p < 0.001$; ****$p < 0.0001$.

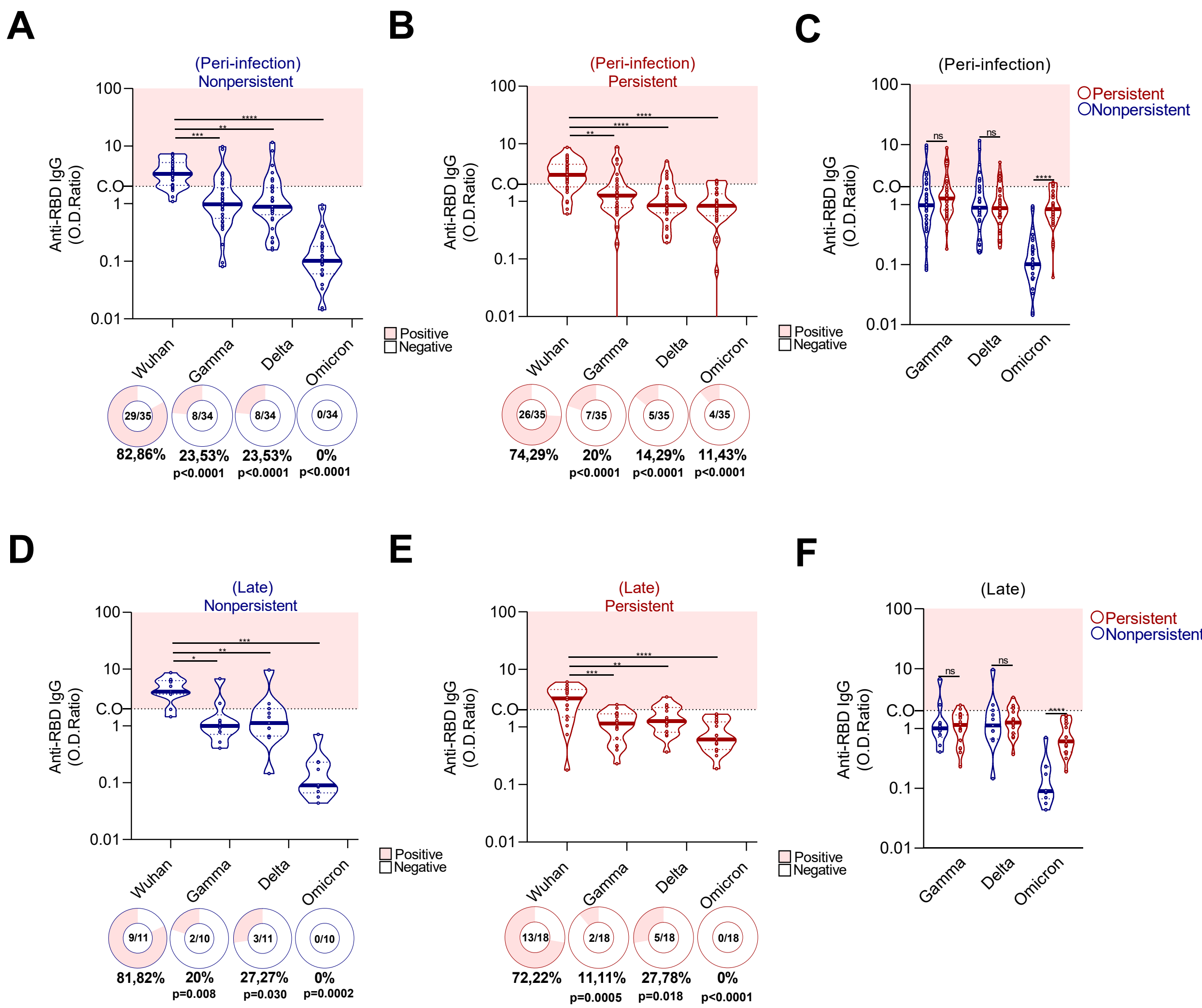

Supplementary Figure 4: Cross-reactive anti-RBD IgG responses to SARS-CoV-2 variants during peri-infection and late convalescence. (A–B, D–E) Relative levels (OD ratio) of cross-reactive anti-RBD IgG against the ancestral Wuhan strain and SARS-CoV-2 variants (Gamma, Delta, and Omicron) in nonpersistent (blue) and persistent (red) individuals during the peri-infection (A–B) and late convalescence (D–E) phases. (C, F) Inter-group comparisons of antibody levels during the peri-infection and late convalescence phases, respectively. Donut charts indicate the proportion of seropositive individuals, with corresponding p-values for group comparisons. Repeated-measures analyses were performed using a mixed-effects model (REML) followed by Tukey's multiple comparisons test. Comparisons between independent groups were performed using the Mann–Whitney U test. Differences in categorical variables, including seropositivity rates, were assessed using Fisher's exact test. Statistical significance is indicated as follows: ns, not significant; *p < 0.05; **p < 0.01; ***p < 0.001; ****p < 0.0001.
